# Lateral optical binding between two colloidal particles

**DOI:** 10.1038/srep38883

**Published:** 2016-12-16

**Authors:** Ming-Tzo Wei, Jack Ng, C. T. Chan, H. Daniel Ou-Yang

**Affiliations:** 1 Bioengineering Program, Lehigh University, USA; 2 Department of Physics and Institute of Computational and Theoretical Studies, Hong Kong Baptist University, Hong Kong; 3 Physics Department, Hong Kong University of Science and Technology, Hong Kong; 4 Physics Department, Lehigh University, USA

## Abstract

An optical binding force between two nearby colloidal particles trapped by two coherent laser beams is measured by phase-sensitive detection. The binding force is long-range and spatially oscillatory. For identical linearly-polarized incident beams, the oscillation period is equal to the optical wavelength. For mutually perpendicular polarizations, a new force appears with half-wavelength periodicity, caused by double inter-particle scattering. This force is observable only with cross-polarized incident beams, for which the stronger single-scattering forces are forbidden by parity.

Upon illumination by a coherent light field, dielectric micro-particles can be coupled together to form a stable array^1^,^2^,^3^. This phenomenon was first demonstrated experimentally by Burns *et al*.^1^,^2^, who recognized its cause to be an optical force resulting from interference of the incident light with light scattered by the particles. In Burns’ experiment, the incident light was focused by a cylindrical lens to form a line of light in the focal plane. The polarization of the light was perpendicular to the orientation of the line, and a pair of dielectric particles were observed to have preferred separation along the line. There are other similar effects where optical binding forces occur along the direction of laser propagation^4^,^5^,^6^,^7^,^8^,^9^, or where standing-wave trapping forces occur in the evanescent wave at a glass–water interface^10^,^11^. For example, N. K. Metzger *et al*.^6^ observed a pair of optically bound dielectric spheres using a dual-beam fiber optic trap. They showed that dielectric spheres modify the field propagation, and the field self-consistently determines the optical forces on the spheres. O. Brzobohaty *et al*.^12^ showed the possibility of multi-stable optical binding in the presence of reflective surfaces. Burns’ effect is called as “lateral optical binding”^13^,^14^,^15^,^16^ to be distinguished from other phenomenon. Although there is evidence for stable colloidal particle self-assembly on a flat solid surface^17^, it is not clear whether a free-standing ordered structure of colloidal particles can self-assemble in two dimensions (2D) when illuminated by linearly polarized light. A numerical study^18^,^19^ showed that a free-standing 2D self-assembly of colloidal particles can occur in a thin-sheet standing wave created by two co-linearly polarized counter-propagating plane waves. Fundamentally different from 1D, in a 2D assembly, the vectors connecting two neighboring particles form various angles with respect to the *E-*field polarization. Thus the force between a pair of particles is a function of both the distance between the particles and the angle between the line connecting the pair and the incident *E-*field.

In this paper, we report direct measurements of the forces between two dielectric Mie particles as a function of the inter-particle distance. We then compare the experimental results with numerical simulation. The line connecting the pair is defined as the X direction. The notations XX, YY, and XY, respectively, mean that the linear polarizations of the *E*-fields incident on the two particles are both in the X direction, both in the Y direction, or one X and one Y, respectively, as shown in Fig. 1 (a–c). The following conditions are required for the experiment: (1) the incident *E*-field polarization for each particle is controlled independently, (2) the two *E*-fields are coherent in phase, and the relative phase of the two *E*-fields is adjustable, (3) the intensities of the *E*-fields on the particles are sufficiently strong that the binding forces in the presence of particle Brownian motion are measureable without the use of high laser power to avoid generating heat^1^,^12^,^20^, (4) the distance between the particles can be varied, and (5) a force sensor capable of measuring femto-Newton forces is present for each particle.

## Experimental procedure

**Figure 1:**
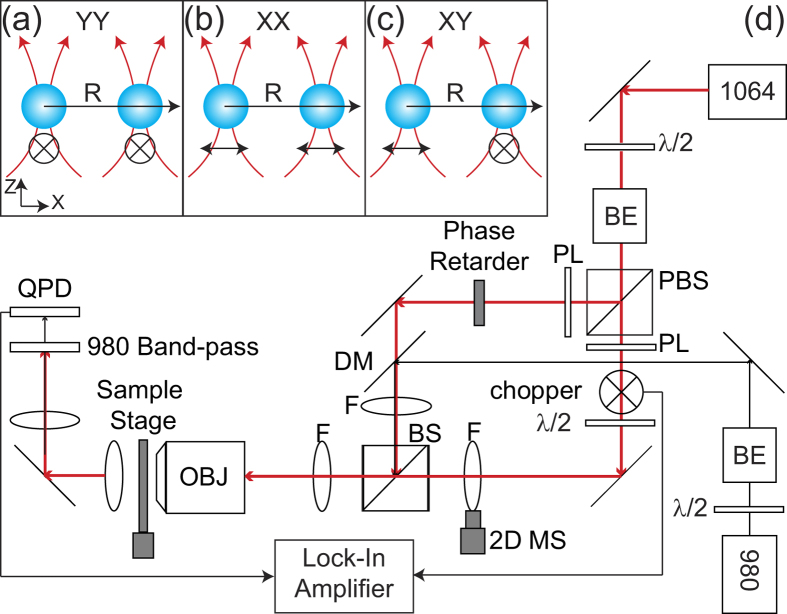
Experimental configurations. In (**a**–**c**), the trapping beams are red and the particles are blue. The polarization directions are indicated in black. The trapping beams propagate in the Z direction. In (**a**) and (**b**), the polarizations of the incident beams are perpendicular (YY configuration) and parallel (XX configuration), respectively, to the X-separation vector of the two particles. *R* is the particle separation. In (**c**), the polarizations of the two beams are orthogonal (XY configuration). (**d**) Diagram of the experimental setup. λ/2: half-wave plate; BE: beam expander; PBS: polarizing beam splitter; PL: polarizer; DM: dichroic mirror; F: lens; BS: beam splitter; MS: movable stage; OBJ: microscopic objective; QPD: quadrant photodiode.

As shown in Fig. 1(d), two optical tweezers produced by a single laser are found to satisfy the requirements for the experiments. A half-wave plate (λ/2), in conjunction with a polarizing beam splitter (PBS), splits the beam into two portions which are passed through two polarizers and are later recombined by a beam splitter (BS) to form the two sets of tweezers. Two 1.5 μm diameter polystyrene particles are positioned in two coherent and independent optical traps, each formed by focusing an IR laser (40 mW, λ = 1064 nm; shown by red lines) with a microscope objective lens (Olympus, PlanFluo, 100X, numerical aperture = 1.3). One of the traps blinks at 31 Hz by use of an optical chopper. Note that the trapped particle under the blinking trapping beam would escape if the frequency of optical chopper is lower or if the trapping area of two traps are overlap. Here, the beam diameter (~0.8 cm) is smaller than the size of chopper aperture (~2 cm). The blinking trap is movable in the X direction by use of a motor-controlled lens in order to change the inter-particle distance. The polarization of blinking trap is altered by the other half-wave plate (λ/2). The other trap is non-blinking and stationary. A second laser (λ = 980 nm; shown by black lines), much weaker than the trapping laser, is used to track the position of the particle in the non-blinking trap. To compare with previous studies via tracking particle fluctuating displacements^1^,^12^,^21^, we use the approach of phase-sensitive detection^22^ and lock-in amplifier to measure optical binding forces between two nearby colloidal particles. A lock-in amplifier, using the blinking frequency and phase as the reference, analyzes the signals from the quadrant-photodiode (QPD) to give the magnitude and phase of the motion of the particle in the non-blinking trap. The particle in the non-blinking trap oscillates with a magnitude proportional to the sum of the optical binding force, a force due to hydrodynamic coupling between the two particles, and an additional optical force due to interference of the two trapping laser beams, all at the blinking frequency. The particle also experiences fluctuating thermal forces. Since the QPD signal from fluctuating thermal forces has a random phase, it is rejected by the lock-in amplifier. Isolating the force due to optical binding from those caused by hydrodynamic coupling and optical interference requires further considerations as discussed below.

## Long-range oscillatory mutual-binding force

**Figure 2:**
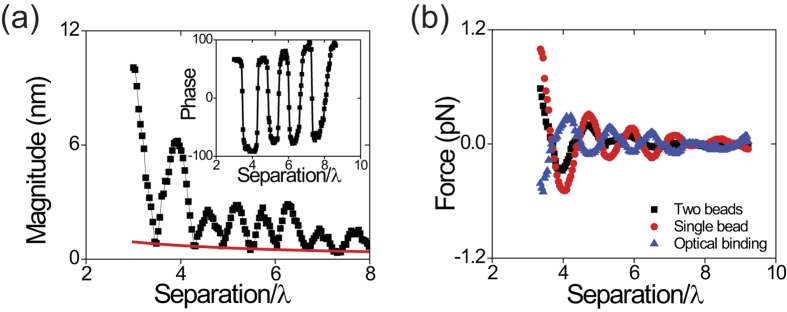
Long-rang oscillatory optical force. (**a**) The magnitude and phase (inset figure) versus center-to-center separation (λ is laser wavelength in the medium ~800 nm) for the motion of the 1.5 μm-diameter polystyrene particle trapped by stationary and non-blinking traps. Here the polarization directions of the two laser beams forming the optical traps are in the YY configuration. The nonzero minima of the data in (**a**) decrease as 1/separation (fitted red curve), resulting from hydrodynamic interactions. By subtracting the red curve, the magnitudes of the displacements (and hence the forces) due solely to optical interactions can be determined. (**b**) Experimental measurements of various types of optical force on the particle in the stationary trap for 1.5 μm polystyrene particles as function of the separation. Black: total optical force with two particles in the dual beam optical trap. Red: optical interference force with a single particle in the stationary trap. Blue: optical binding force obtained by subtracting off the interference force (red dots) from the total optical force (black dots).

Combining optical tweezers with known optical spring constants as force sensors and phase-sensitive detection^21^,^22^,^23^, we measure the forces between the two particles. Using a lock-in amplifier, we measure the magnitude and phase (relative to that of the blinking light) of the displacement of the particle in the non-blinking trap as a function of the distance between the particles as shown in Fig. 2(a). Due to the time-periodic force produced by the blinking tweezers, both particles in the optical traps oscillate at the blinking frequency. The motions of the two particles are due to both optical and hydrodynamic coupling^24^,^25^. Here, the hydrodynamic coupling is shown by the nonzero minimum displacement which indicate non-binding force at zero-phase shift as shown in the inset figure of Fig. 2(a). The line connecting the minimal points is fit by 1/separation^24^,^25^ as shown in red in Fig. 2(a). We obtain the particle displacement due to the optical force by subtracting from the displacements due to hydrodynamic coupling.

Not all the optical forces on the particles are due to optical binding. When two optical tweezers are near each other, each exerts forces on both particles, and the strength of the optical cross-talk increases with decreasing particle separation. Since two overlapping coherent optical fields interfere, the particles experience a force due to the interfering field. We determine the force due to cross-talk by measuring the force on the particle in the non-blinking trap when the blinking trap contains no particle, thus eliminating all optical-binding and hydrodynamic forces. The pure binding force is then found by subtracting the optical cross-talk force from the total optical force as shown in Fig. 2(b).

To compare the experimental results with numerical simulation, we compute the optical fields^26^,^27^ by first using a Debye integral to calculate the highly localized incident optical field of an optical tweezers^28^,^29^,^30^, and then calculate the scattered field^18^,^28^,^31^ from a Mie particle in the optical tweezers by use of the generalized Mie theory. We then integrate the Maxwell stress tensor^18^,^31^,^32^,^33^ to calculate the forces on the particles, the polarizations, and the relative phase of the two trapping beams as a function of the inter-particle separation.

**Figure 3:**
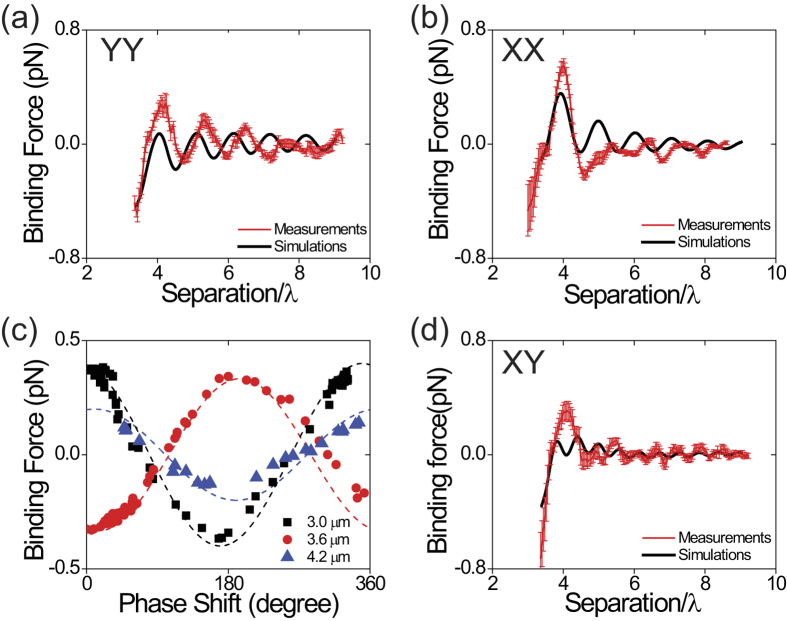
Comparison of experimental results and theoretical calculations. Experimental measurements (red points) and calculation (black lines) of the optical binding force for the polarization configurations (**a**) YY and (**b**) XX as a function of the center-to-center separation (λ is laser wavelength in the medium ~800 nm) between two 1.5 μm polystyrene spheres. (**c**) Binding forces for the YY configuration as a function of the relative phase between the two optical traps. Black, red, and blue correspond to separations of 3.0, 3.6, and 4.2 μm, respectively. The dotted lines are guides to the eye illustrating the sinusoidal nature of the curves. (**d**) Experimental data (red points) and theoretical simulation (black line) of the binding force for the XY orthogonal polarization configuration as a function of separation between two 1.5 μm polystyrene spheres. The means and the standard deviations were obtained by repeating each experiment 10 times under identical experimental conditions.

Figure 3 shows both the measured and calculated binding forces for the YY (Fig. 3(a)) and XX (Fig. 3(b)) configurations *vs.* the center-to-center separation between two 1.5 μm-diameter polystyrene spheres. The theoretical calculation shows that the spatial periodicity of the binding force for both the YY and XX configurations are equal to the laser wavelength in the medium (~800 nm). However, the experimental data show the spatial period to be longer than the theoretical prediction. This discrepancy is due to an experimental effect: the optical path-length changes when the distance between the particles is altered, introducing an additional phase shift to the retardation between the *E*-fields at the locations of the two particles.

To demonstrate that the spatial oscillation of the binding force is caused by a phase delay, we use a phase retarder (ARCoptix, Switzerland) to measure the force as a function of the relative phase between the two trapping beams at fixed relative positions. The force *vs.* relative phase for the YY configuration is shown in Fig. 3(c). Black, red, and blue symbols correspond to particle separations of 3.0, 3.6, and 4.2 μm, respectively; the data show that the optical binding force at a fixed particle separation varies sinusoidally with the relative phase, and the maximum magnitude of the force decreases with separation, as expected.

### Self-binding force with half-wavelength periodicity

The magnitude of the XY binding force (Fig. 3(d)) is smaller than XX and YY by a factor of two, and the XY binding force shows different characteristics from XX and YY. The spatial periodicity of the XY binding force is ½ wavelength, and the force at a fixed particle separation is independent of the relative phase of the two beams. Conventional optical binding^1^,^2^ cannot occur in the XY configuration, since simple binding forces of the type seen with XX and YY polarizations are forbidden by parity. The optical force in the XY configuration is caused by interference of the *E*-field of the trapping beam with that scattered first from the particle in the trap to the neighboring particle, then re-scattered back to the original particle. Since this doubly scattered wave travels a distance 2*R*, the phase delay between the incident and the doubly-scattered wave is 2*kR (k* is the wave number of the laser beam in water and *R* is the particle separation), which gives the optical force a spatial oscillation with period ½ wavelength.

Compared with the XX or YY “mutual-binding” force proposed by Burns *et al*., the XY “self-binding” force is a higher-order effect in the sense that it is induced by doubly scattered waves, thus its magnitude is smaller than that produced in the XX or YY configurations. The only reason this self-binding force is detectable in the XY configuration is due to XZ-plane reflection symmetry disallowing the first-order mutual-binding force. It is worth noting that the self-binding force exists in the XX and YY configurations as well, but in these configurations the self-binding forces are masked by the lower-order and stronger mutual-binding force.

A simple argument why mutual binding is forbidden for the XY configuration is as follows. The system consists of two plane-incident waves propagating in the Z direction, one X-polarized and one Y-polarized. The waves illuminate a high-numerical-aperture objective lens and are then being focused onto two particles. Upon an XZ-plane reflection of the entire system, the particles and the lens are unchanged, as they possess XZ-plane symmetry. The sign of the X-polarized plane incident wave remains unchanged, while the sign of the Y-polarized plane incident wave reverses. Since the mutual binding force is proportional to the product of the fields induced by the X- and Y-polarized plane incident waves, the mutual binding force changes sign upon an XZ-plane reflection. Since Maxwell’s equations are invariant upon mirror reflection, the mutual binding force should be invariant under such a reflection. Thus, on one hand the mutual binding force changes sign, and on the other it must be invariant. The contradiction can only be resolved if the mutual binding force is zero. In short, a mutual binding force between two particles trapped by two beams in the XY configuration cannot exist. This result is independent of the particle size and is unaffected by the detailed field distribution.

## Symmetry argument for self-optical binding

A mathematical treatment of the above symmetry argument is given. We assume two strongly focused trapping beams (one X-polarized and one Y-polarized), whose centers are separated along the X-direction and each trapping a spherical particle. The particles can be different in size, composition, or other aspect, as long as their morphology is symmetric upon a reflection on XZ-plane. The optical force is simply the linear superposition of the force produced by the X-polarized beam and the Y-polarized beam independently. The phenomenon is surprising because one generally expects that the two beams will interfere, especially for different sized particles and when the polarizations of the two beams are not orthogonal due to the existence of Z component electric induced by strong focusing.

Consider two spheres, A and B, located on the X-axis and separated by a center-to-center distance *R*. Spheres A and B are trapped separately by focused beam 1 and 2 (both Z-propagating), respectively. Focused beam 1 and 2 are X- and Y-polarized, respectively. Consider the X-component of the force, *F*_*x*_, (which is the binding force) acting on sphere A. Now suppose we take a mirror reflection about the XZ-plane. The symmetry of the focused trapping beam upon mirror reflection on the XZ-plane is the same as that of the incident beam plus that of the geometry. Since the geometry is invariant upon XZ-plane reflection while the incident X- and Y-polarized beams have an even and odd parity, respectively, then the field of focused beam 2 changes sign (equivalent to a change in phase of *π*) while that of focused beam 1 is invariant. We note that since Maxwell’s equations are invariant upon mirror reflection, the system after the mirror reflection is still a physical system. *F*_*x*_ is invariant upon this symmetry operation. Accordingly


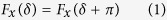


where *δ* is the relative phase between the two trapping beams. We shall show, by inserting Eq. (1) into the Maxwell stress tensor, that the condition Eq. (1) implies *F*_*x*_ is independent of *δ*.

To consider the optical force acting on sphere A, the total electromagnetic field consists of the two incident trapping beams plus the scattered waves from both beams, i.e.


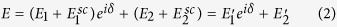


where 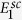 and 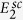 are the scattered field due to the illumination of beam 1 and 2, respectively, *e*^*iδ*^ represents the relative phase between the two beams, and 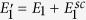 and 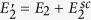. Similar notation is used to label the magnetic field. The time averaged optical force acting on sphere A is given by


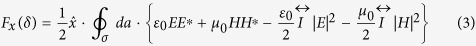


where σ is the surface of sphere A. By substituting Eq. (2) and its magnetic analog into Eq. (3), we obtain


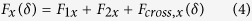


where


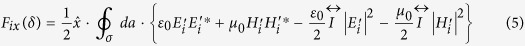


is just the optical force when there is only the *i*-th beam (other beams are absent), is independent of *δ*, and


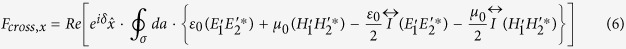


is the cross term between beam 1 and 2, which is dependent on *δ*.

Substituting Eq. (4) into Eq. (1) and noting from Eq. (6) that 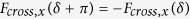, we obtain


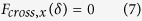


Consequently,


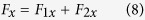


which is independent of *δ*. From Eq. (8), it is clear the two beams exert forces independently. The total optical binding force is just the linear superposition of the forces that would have been produced by the first and second beam independently. This is true independent of particle size, compositions, and morphology.

## Conclusion

In conclusion, this article reports an experimental and theoretical investigation that generalizes the lateral optical binding observed first by Burns *et al*.^1^,^2^. Here, dual coherent optical tweezers are used to generate lateral optical binding forces between two colloidal particles as a function of inter-particle spacing. The binding forces are long-range and oscillatory in space. Three *E*-field polarizations relative to the direction between the particles are examined. For *E*-fields polarized perpendicular (YY) or parallel (XX) to the inter-particle direction, the binding forces oscillate with a periodicity equal to the wavelength of the light, consistent with the finding by Burns *et al*. A new binding force was discovered for the XY configuration where the incident *E*-field polarizations at the two particles are mutually perpendicular. This binding force has a spatial periodicity equal to half the light wavelength. Calculations based on Mie theory and the Maxwell stress tensor indicate that the new binding force is caused by double scattering of light from the two particles. We remark that if metallic particles^34^,^35^,^36^ are used instead of dielectric particles, the self-binding phenomenon would be enhanced. However, metallic particles are more difficult to trap. In contrast to the classical “mutual-binding” of Burns *et al*.^1^,^2^, which is caused by single scattering, the new binding force is a second-order effect; it is observable only because the first-order mutual-binding is forbidden by parity in the XY configuration. Since the partner particle serves only as a back-scatterer, we refer to the new optical binding as a “self-binding” force, which is potentially responsible for the attraction of coherent light-illuminated particles to nearby solid walls^11^.

## Methods

### Theoretical modeling

Computation of the optical force involves the modeling of the strongly focused trapping beams using the vector Debye integral, Mie scattering theory for the scattering problem, and Maxwell stress tensor formulation for the optical force. The incident beam is modeled by the generalized Mie scattering theory together with the electromagnetic generalization of Debye integral^28^,^29^,^30^. In this approach, the focusing of the incident beam by the high numerical aperture objective lens is treated by geometrical optics, and then the focal fields are obtained by using the angular spectrum representations^26^. This is legitimate as the size of the lens is much greater than the wavelength. However, geometrical optics does not work all the way down to the focus. In the vicinity of the beam focus, the vector Debye integral must be invoked to relate the far field obtained from geometrical optics to the near field in the focal region. The water/glass/oil interface in the optical trap introduces spherical aberration, which is taken into account using the approach of Torok *et al*.^27^. This method is proven to agree quantitatively with experiments^31^,^32^,^33^. The generalized Mie theory is used to solve Maxwell’s equations for the electromagnetic fields scattered by spherical particles^18^. The incident field to, and the scattered field from, the spheres are expanded in vector spherical wave functions, which are the quasi-normal modes for the spheres. The expansion coefficients are obtained, with the help of the vector translation and addition theorems, by matching the standard electromagnetic boundary conditions over the surface of all spheres. Then, we calculate the time-averaged (total) optical force, *F*, acting on a particle via a surface integral of the time-averaged Maxwell stress tensor 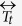 over the particle’s surface, *S*: 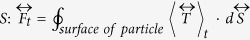 and 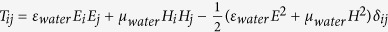, where *E* and *H* are the electric and magnetic fields, and *ε*_*water*_ and *μ*_*water*_ are the relative permittivity and permeability of the water, respectively. We note that the expression is valid in water since water is incompressible. In our numerical model, multiple scattering between the spheres are taken fully into account. It can be considered as exact within classical electrodynamics up to numerical truncation errors, in the sense that there is no approximation. Once the total force for a pair of spheres is calculated, we then subtract from the force for a single sphere to obtain the pure optical binding force.

## Additional Information

**How to cite this article**: Wei, M.-T. *et al*. Lateral optical binding between two colloidal particles. *Sci. Rep.* **6**, 38883; doi: 10.1038/srep38883 (2016).

**Publisher's note:** Springer Nature remains neutral with regard to jurisdictional claims in published maps and institutional affiliations.
